# Bacteriophage Assembly

**DOI:** 10.3390/v3030172

**Published:** 2011-02-25

**Authors:** Anastasia A. Aksyuk, Michael G. Rossmann

**Affiliations:** Department of Biological Sciences, Purdue University, West Lafayette, IN 47907, USA; E-Mail: aaksyuk@purdue.edu

**Keywords:** bacteriophage, assembly, macromolecular complex, genome packaging, maturation

## Abstract

Bacteriophages have been a model system to study assembly processes for over half a century. Formation of infectious phage particles involves specific protein-protein and protein-nucleic acid interactions, as well as large conformational changes of assembly precursors. The sequence and molecular mechanisms of phage assembly have been elucidated by a variety of methods. Differences and similarities of assembly processes in several different groups of bacteriophages are discussed in this review. The general principles of phage assembly are applicable to many macromolecular complexes.

## 1. Introduction

**Figure 1.**
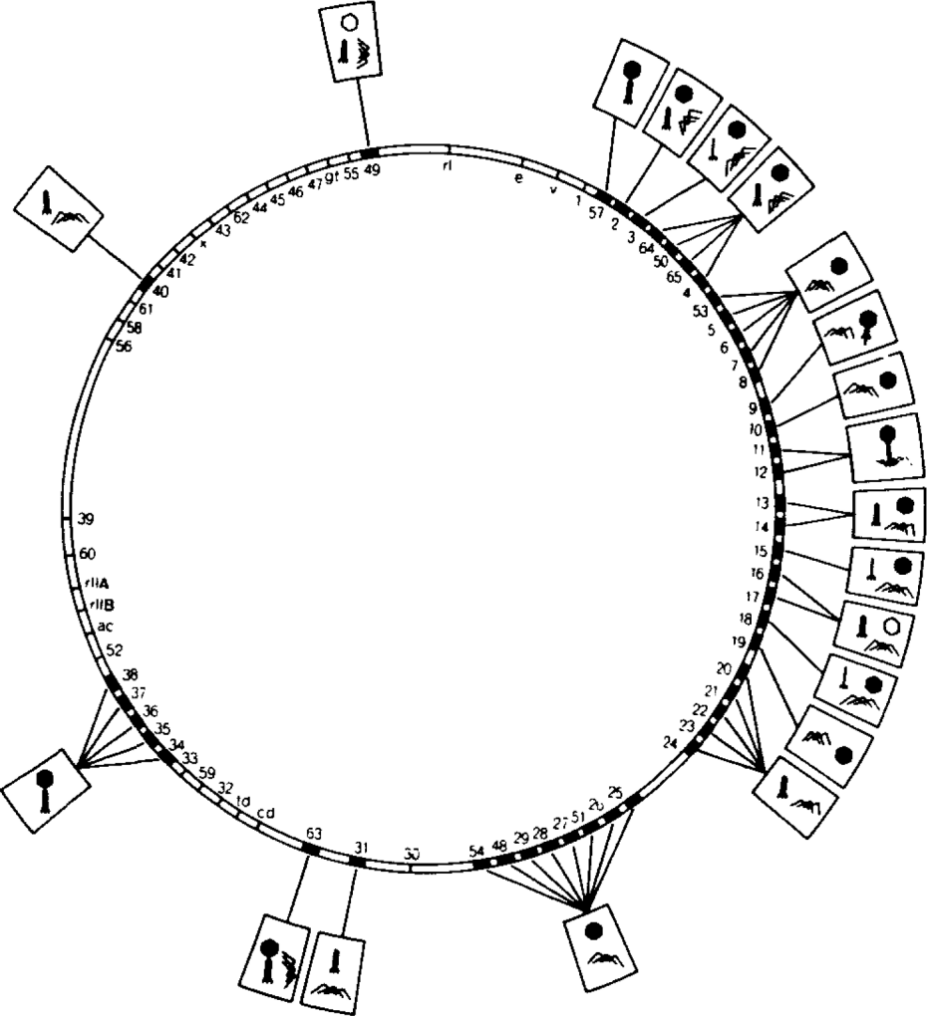
A simplified bacteriophage T4 genome map showing the effect of mutations in morphological genes on virion assembly. Boxed symbols attached to a particular gene show the phenotype, arising from the defects in that gene. Reprinted from Wood *et al.* [1]; with permission. Copyright for this figure is owned by the Federation of American Societies for Experimental Biology. This image may not be reproduced in any way without explicit permission from the copyright owner.

How proteins and nucleic acids assemble, often spontaneously, into structurally well-defined three-dimensional objects is an intriguing question. The limited size of the phage genome and the multicomponent composition of bacteriophages make them well suited for assembly investigations. Genetic manipulation of phages has made it easy to observe the effects of gene inactivation on protein-protein association, providing information on the sequence of assembly processes (Figure 1; [1]). Over the past fifty years, mutational, biochemical and biophysical analyses, X-ray crystallography, NMR, cryo-electron microscopy (cryo-EM), thin sectioning and single molecule methods have been used to study bacteriophages [2–15]. This review will describe what has been achieved and will contemplate what still needs to be accomplished, focusing mostly on dsDNA tailed phages (Table 1).

There are substantial similarities in the assembly processes of all types of viruses, and of various cellular complexes. For example, most bacteriophages as well as herpesviruses, adenoviruses, poxviruses and the giant mimivirus make an empty protein shell that is subsequently packaged with the viral genome [4,5,16–21]. The majority of viruses undergo proteolytic cleavages during assembly that are often essential to trigger the next assembly step [22,23]. Additionally, the capsid protein fold common to dsDNA tailed phages also occurs in herpesviruses [24], as well as in bacterial molecular compartments [25]. Moreover, the bacterial injectosome, the hook of flagella and phage tails all use molecular ruler proteins in order to assemble a complex of correct length [26]. Nevertheless, even within the same group of phages, there are notable differences in the assembly pathway, size and symmetry of the capsid and tail, positions of individual genes in the genome, the number of structural proteins and whether or not they are cleaved during assembly [27]. In this review we discuss only those groups of phages for which a significant amount of knowledge on assembly has been accumulated. Thus, for instance, we make no mention of *Corticoviridae* and *Plasmaviridae* families of phages.

## 2. dsDNA Tailed Phages

All tailed bacterial viruses are distinguished by a special organelle, called a tail, which contains cell recognition proteins and triggers DNA release from the head. Tailed phages form the *Caudovirales* family, which is subdivided into phages with short tails (*Podoviridae*), phages with long non-contractile tails (*Siphoviridae*) and phage with contractile tails (*Myoviridae*). Viruses with similar morphology are found in archaea, suggesting that tailed phages appeared before the separation of the domains of life [28,29]. The size of the tail ranges from a small protein complex of about 40 proteins in *Podoviridae* phages to about 300 proteins in *Myoviridae* tails. The length of the tail varies from about 300 Å in P22 to about 4550 Å for bacteriophage G [30–32]. Although tails are not commonly observed in eukaryotic viruses, algal viruses have been reported to have similar structures [33] and a tail-like unique vertex was demonstrated for PBCV-1 [34].

### 2.1. Head Organization

Many bacterial and eukaryotic viral capsids have similar organization. Multiple copies of viral capsid proteins are frequently assembled into icosahedral shells, as was predicted by Crick and Watson [35] and later confirmed by Caspar [36]. A minimum of 60 copies of the capsid protein is necessary to make an icosahedral particle, in which case all the subunits have identical environments. A bigger capsid can be made by placing several monomers into the icosahedral asymmetric unit with “quasi-equivalent” environments, making the total number of capsid proteins a multiple of 60. For example, if there are T (the triangulation number) quasi-equivalent positions, there are T × 60 capsid proteins in a capsid [37]. A T = 1 particle consists of twelve pentameric capsomers, whereas a capsid with a higher T number contains both pentameric and hexameric capsomers. Not all T values are permissible (although some disallowed T numbers can occur in practice [38]) as some T numbers cannot be arranged with quasi-symmetric environments. However, for each virus there is usually a dominant capsid organization. This implies that virus assembly must ensure that the capsid proteins form a unique structure with a specific T number. A different way of increasing capsid size is assembling an elongated or prolate head, by inserting an additional cylindrical section between the two icosahedral end-caps. Such a structure is described by two triangulation numbers: a T number is used for the terminal caps and a Q number for the cylindrical section. Prolate heads occur in several genera of plant, fungal and animal viruses [39], but are more common among bacteriophages, where they are observed in about 15% of studied viruses [28]. As was pointed out by Moody [40], elongation of the head in one direction does not affect receptor binding sites in bacteriophages, because only the vertex where the tail is attached to the head is functional for cell binding. In a prolate head there are two types of five-fold vertices, the ten surrounding the cylindrical section and the two five-folds at the center of the caps. These differences would affect receptor attachment and uncoating in viruses that otherwise use any of the five-fold vertices during infection (e.g., picornaviruses) perhaps explaining the rare occurrence of prolate heads among eukaryotic viruses [40].

Most dsDNA tailed phages assemble their capsid from multiple copies of one capsid protein. However, bacteriophage T4 encodes two capsid proteins, one forming the pentameric capsomers, consisting of gp24, and the other hexameric capsomers, consisting of gp23. Although both are present in the wild-type T4 phage, a mutant gp23 can form both hexameric and pentameric capsomers, producing a capsid that contains only one type of capsid protein [41–43]. These two proteins share 21% sequence similarity and probably occurred as the result of gene duplication [42,44]. Many eukaryotic viruses, like herpesvirus, adenovirus, picornaviruses, PBCV-1 and others, code for more than one capsid protein [45]. Although the presence of several different proteins requires a larger viral genome, it might provide some evolutionary advantages. For example, the problem of accommodating quasi-equivalent environments in the vicinity of pentameric vertices is mitigated. Additionally, mutations would be easier to accommodate, as they would affect only certain positions in the capsid [23].

### 2.2. Formation of the Prohead

An empty shell, called the prohead or procapsid, is usually a required stable assembly intermediate which is then packaged with DNA. The difference in the structure of proheads and mature heads is discussed in Section 2.4. Prohead assembly is not unique to bacteriophages, but also occurs in many other viruses such as adenoviruses, herpesviruses, poxviruses and the giant mimivirus [18–21]. The similarities between tailed phages and herpesviruses are even more striking as they were shown to share the same capsid protein fold [24], termed a HK97-like fold, which was first found in bacteriophage HK97 [46]. However, some phages have domains in the capsid protein structure that are not part of the HK97 fold. Such domains are structurally distinct in different phages, represented by BIG2-like domain in phi29 [47], a chitin-binding-like domain in T4 [44] and an insertion domain in P22 [48–50]. In T4 and phi29 these insertion domains stabilize the capsid, by bridging the neighboring molecules within one capsomer (T4) or between neighboring capsomers (phi29). Although the capsid protein of HK97 does not have an insertion domain, the capsid is stabilized through a network of covalent cross-links [46]. Bacteriophage T7 does not have an extra capsid domain, but 10% of its capsid proteins have C-terminal extensions of 52 residues, which arise from a frameshift during translation [51]. Although complementation showed that T7 capsid without the larger protein is as stable as the wild-type virus, this frameshift is conserved in the related phage T3 [51].

A capsid protein by itself is not capable of ensuring the correct geometry of the capsid shell, requiring an additional scaffolding protein for prohead assembly [8,52,53]. Most phages have a separate scaffolding protein gene, with the exception of HK97 and T5 that have scaffolding domains, or delta-domains, within capsid protein sequences [54–56]. In assembled procapsids, scaffolding proteins form a core inside the prohead, which does not have the icosahedral symmetry of the outer capsid shell. Many phage scaffolding proteins, including those of P22, phi29 and SPP1, form dimers and tetramers [57–59], increasing the local concentration of capsid proteins and therefore acting as an entropic sink and promoting association of coat proteins [8,53]. Without scaffolding most capsid proteins form aberrant structures. In the case of P22, the assembly of aberrant particles proceeds ten-times slower than head assembly in the presence of the scaffolding protein [60]. A mutation in the scaffolding protein of phi29 leads to the formation of isometric particles instead of prolate [61]. In the absence of scaffolding proteins, capsid proteins of T4 and lambda can assemble into long cylindrical structures [62–64]. Similarly, bacteriophage T5 capsid protein can form open tubular assemblies under certain conditions, even though the scaffolding domain is a part of the capsid protein sequence [56]. Assembly of bacteriophage T7 into polycapsids occurs in the presence of scaffolding protein, but only when the ratio of scaffolding protein to the capsid protein is 0.11:1 [65,66]. The resulting polycapsids consist almost entirely of head protein [66].

Both the phi29 scaffolding protein and the C-terminal portion of the P22 scaffolding protein have a helix-loop-helix structure [58,67]. Moreover, the sequence alignment suggests that the N-terminal delta domains of HK97 and T5 would have a similar motif [58]. The binding sites of P22 scaffolding protein on the capsid were determined by electron microscopy, but the arrangement of the scaffolding protein inside the capsid could not be visualized due to icosahedral averaging [57,68,69]. The reconstruction of the phi29 prohead assuming no symmetry showed a cage-like scaffolding protein density inside the capsid, organized in several shells with different symmetry [58]. In contrast to the majority of phages that code for one scaffolding protein, the bacteriophage T4 capsid assembly requires six different scaffolding, or core, proteins [70,71]. Co-expression of these proteins results in formation of tubular polycores which are unique to T4 [72]. Although it was previously shown that the core of T4 has six-fold symmetry, a later re-examination indicated that polycores could have six-, eight- or ten-fold symmetry [73]. How exactly scaffolding proteins influence the shape of the head is unknown.

The third essential component of head assembly is a portal protein, also called a connector, which is situated on one pentameric vertex of the capsid. As these names suggest, portal or connector is required for DNA entry and release, as well as for attachment of the neck proteins to the head. Additionally, the phage head assembly is probably initiated from the portal vertex by copolymerization of the scaffolding and capsid proteins [3,15,74]. In bacteriophage T4, the ellipsoidal cores are formed in the absence of the capsid protein [75], whereas in many other phages the scaffolding proteins cannot assemble without the presence of capsid proteins. Assembly initiation in bacteriophage T4 additionally requires a membrane scaffolding protein, which interacts with the connector protein [76]. The membrane association of proheads during the assembly was not demonstrated for other phages.

The recombinantly expressed portal proteins were shown to assemble into different oligomers, but in all structurally characterized phages the portal protein forms a dodecameric ring [10]. Therefore, to ensure proper prohead assembly the portal has to co-assemble with the capsid and scaffolding proteins [77,78]. The outer diameter of portal protein rings ranges from 140 to 170 Å and the protein molecular weight ranges from 36 kDa (phi29) to 83 kDa (P22) [74]. Electron microscopy and X-ray crystallography have shown that the basic morphology of the portal ring is similar in all known tailed phages [79–82]. Some phages, like P22, HK97 and T7, can form prohead-like structures in the absence of portal proteins. However, these virus-like particles cannot package DNA and represent a dead end assembly. It was noted earlier that aberrant particles often form slower than the viral precursors, indicating that connector and scaffolding affect not only the accuracy but the kinetics of the assembly process [60,83,84].

### 2.3. dsDNA Packaging

Once the head is assembled, the packaging complex binds and utilizes ATP hydrolysis to translocate the genome into the head [5,13,85,86]. Because of a symmetry mismatch between the dodecameric portal ring and the five-fold capsid vertex, it was suggested that the portal ring is rotating during packaging [87]. Later single-molecule spectroscopy of the phi29 connector [88] and studies on T4 connector in which its motion was restricted by binding to the capsid [89] showed that connector rotation does not accompany packaging.

The initiation of DNA packaging depends on how viral DNA was replicated. Many phages produce head-to-tail multimers, or concatemers of DNA, and these are used as a packaging substrate in T4, lambda, and P22. In that case, the ATPase packages the length of the genome (or more in some phages) and cuts the concatemer. Hence, the term “terminase” is used for the packaging ATPase. This enzyme is also called a large terminase to distinguish it from the small terminase, which does not cut the DNA. The small terminase modulates the activity of the large terminase and is involved in packaging initiation [5,90]. The crystal structure of a small terminase from a *Podoviridae* phage Sf61989 was solved by X-ray crystallography, suggesting it could form a ring below the large terminase [91]. Bacteriophage phi29 does not have a small terminase, but it utilizes a unique viral encoded structural RNA, called p-RNA [92,93]. Some phages, like Mu, N4 and phi29, do not generate a concatemeric DNA [5]. Phages that utilize concatemeric DNA package more than one length of the genome, or a headful. The determination of when the head is full is not well understood. There are known mutations in T4, P22 and SPP1 that demonstrate the interplay between the terminase and portal protein ring [74,94–97]. Therefore, conformational changes in the connector are thought to provide the signal for the terminase to cut the DNA and dissociate from the head [5,74,82]. Single molecule studies of the phi29 packaging motor [98,99] and the crystal structure of T4 terminase [100], as well as the structure of the dsRNA packaging enzyme from phage phi12 [101], have formed the basis for several packaging mechanisms [13,102–104].

The packaged genome inside the phage head is wound into a spool-like structure such that several layers of dsDNA are visible in electron micrographs of individual virions as well as in cryo-EM image reconstructions of phage heads [10,105]. There are several proposed models for the formation of the spool structure [5,12,106,107]. The DNA end that is packaged first is likely associated with the inside surface of the capsid, whereas the end that is packaged last is probably the first to be ejected [5]. Strong binding of DNA to the connector was shown for phage SPP1 [97] and both ends of DNA were localized within or near the portal in bacteriophage T4 [108].

### 2.4. Head Maturation

During or before packaging the scaffolding proteins either exit from the capsid (P22, phi29) or are proteolytically cleaved by phage encoded protease (HK97, T4). The recycled P22 scaffolding protein can be reused four more times in the assembly process [109]. Lambda scaffolding protein can also exit from the capsid without being cleaved, as was shown for the phage with a genetically inactivated protease [110]. The release of scaffolding proteins could be induced by their interaction with DNA, possibly through a leucine-zipper motif, identified in the scaffolding protein of phi29 [58]. The exit of the scaffolding proteins and the packaging of DNA initiate maturation, involving a large structural transition of the prohead and resulting in a bigger, angular and more stable head with a thinner shell [3,6,8,9,14,40,64]. In contrast to mature heads, proheads can dissociate into subunits at low concentration [111], which could allow proofreading and correction of misassembled intermediates. Additionally, the thick shell of the prohead might make it easier to control the curvature during assembly [40]. In some phages, like HK97 and T4, cleavage of the capsid and scaffolding proteins precedes the expansion, but the cleaved-unexpanded intermediates are short-lived. The viral protease of T4 cleaves about 3,000 peptide bonds per virion [70]. Cleavages of capsid protein can affect the thermodynamic stability of the capsid through changes of quaternary interactions [23] and could also influence the kinetic stability of capsid protein as shown for self-cleaving enzymes [112,113]. In bacteriophage lambda, in addition to the capsid protein cleavage, the connector protein is also cleaved [114]. Although this cleavage is not essential for assembly, it might play a role during DNA ejection [23]. *In vitro* treatment of proheads with denaturants can also trigger maturation, probably through unfolding of the domains that are cleaved by protease *in vivo*. Maturation intermediates of HK97 proheads and T4 polyheads have been trapped *in vitro* [115–119]. Head expansion is probably initiated at one end by the portal protein [120] and is then propagated through the prohead. Such a wave was captured in a giant capsid of phage T4, for which several different maturation states were observed along the axis of the head [121]. The head expansion leads to a 50% increase of the head volume and a change in the appearance of the hexameric capsomers, which in most phages have two-fold rather than six-fold symmetry in the prohead [105,118,122,123]. In phage P22 skewed capsomers have central holes, through which the scaffolding proteins could exit [49,123]. Although detailed information about the head expansion during maturation is derived mostly from the work on HK97, it is likely to be applicable to other tailed phages, all of which have the same capsid protein fold. Comparison of the prohead and the mature head crystal structures of HK97 showed that the capsid proteins are probably trapped in a distorted form. During maturation of HK97, the cross-linking reaction drives the procapsid into a metastable state, where the capsid protein refolds into its lower energy conformation after the delta-domain has been cleaved [14]. Although the cross-linking is unique for HK97, the binding of capsid stabilization proteins, represented by gp soc in T4 and gpD in lambda, could act similarly during maturation and promote the head expansion [14,124–127]. Both gp soc and gpD can only attach to matured capsids, but have different effects on capsid stability. In the absence of gpD, lambda capsids cannot package the full genome [128], whereas gp soc is only required in the extremes of pH and temperature [124].

In the course of head assembly many phages incorporate minor or pilot proteins into the head. These are usually present in low copy numbers (less than 12 subunits) and are nonessential for the formation of the structure, but crucial for infectivity of the virion. There are three minor proteins in P22, which modulate DNA ejection [52,129] and were thought to localize in the shaft above the connector [82]. However, the shaft density was later reassigned to be part of the connector [50]. A more elaborate shaft structure, called the inner core, is present in the capsid of bacteriophage T7 [65,105,130] and consists of a ring of proteins with twelve-fold symmetry immediately above the connector, followed by an eight-fold and a four-fold symmetric protein rings. A difference in the core structure before and after DNA packaging is perhaps important for the release of terminase after packaging. A similar signal could be propagated by a conformational change of the connector [82,131].

### 2.5. Head Assembly Completion

After the head is packaged with the DNA, the terminase complex is substituted by neck proteins, which together with the connector form the “gatekeeper” complex [81], preventing premature DNA leakage from the head and possibly initiating DNA exit upon attachment to the host bacteria [132]. In *Siphoviridae* and *Myoviridae* phages there are two types of neck protein, each making a ring below the portal. The neck proteins that form a ring closest to the connector have similar structure in *Siphoviridae* phages SPP1 and HK97, but have a different fold in lambda. However, the neck proteins forming the second ring have similar folds in SPP1 and lambda [132–136]. Additionally, a *Myoviridae* prophage was identified that has structurally similar neck proteins to those of *Siphoviridae* [135]. A related structure of the tail-binding platform among *Sipho*- and *Myoviridae* phages indicates a common tail binding mechanism and suggests an evolutionary relationship, as well as a possibility that there once existed a phage which could attach two different tail structures.

Phage neck proteins, as well as several other structural proteins, like gpD of lambda, have large (more than 60%) unstructured regions. Naturally disordered proteins that participate in protein-protein interactions are abundant in cells and by becoming folded can influence the sequence of assembly [137]. Additionally, it has been shown that a small disordered protein, which does not have a hydrophobic core, can donate a large surface area to the binding interface. On the other hand, an ordered protein has to be much larger to donate an equivalent surface area to complex. The presence of unstructured proteins inside the cell was suggested to prevent overcrowding [138], whereas for the virus it could play a role in keeping the genome size small.

### 2.6. Tail Assembly

After the completion of the head assembly, the tail proteins of *Podoviridae* phages are sequentially attached to the capsid [139,140]. However, for *Sipho-* and *Myoviridae* phages, there is a separate tail assembly branch to the assembly pathway, allowing the preformed tail to bind the head via the neck proteins [15]. In the case of T4, this is followed by an attachment of the preassembled fibers [71,141]. Although the tail and the head assemblies occur independently, within each pathway protein association follows a strict order, implying that the third component does not bind until the first two proteins form a complex. Likewise, if an assembly component is absent, the assembly is stalled, and all the proteins that would be added after the missing component should have assembled will remain free in solution. Such a sequential assembly was shown, for example, for the distal part of the T4 contractile tail, called the baseplate [142–144]. In such processes the monomers are added to a growing complex and are not wasted on incomplete intermediates [15].

The sequential attachment of proteins can be controlled by different mechanisms. One of the mechanisms, very common in protein assemblies, is called conformational switching. Such a process occurs when a protein structure changes upon attachment to an initiator complex and often involves refolding of some part of the structure, for example a loop-to-helix. Conformational switching is observed in viral capsids with T numbers higher than one, when a capsid protein has to adopt several quasi-equivalent conformations [145–147]. The sequence of assembly can also be controlled though formation of composite binding surfaces, created by more than one protein. In such a situation the portion of the surface donated by a single protein is insufficient for stable attachment of an assembly component [15,148].

The tail assembly in *Siphoviridae* and *Myoviridae* phages starts from the initiator complex, which forms the absorption device of the phage at the distal end of the tail. The size of this complex ranges from six proteins in *Siphoviridae* phage lambda or eight in *Myoviridae* phage Mu [149,150] to about 150 in the baseplate of *Myoviridae* phage T4 [71,151]. In addition to priming the tail assembly, baseplate complexes undergo structural changes during infection that involve large motions of the component proteins, as was shown for baseteriophage T4 [152] and Lactococcal *Siphoviridae* phage p2 [153]. During tail assembly, baseplate initiates polymerization of the cylindrical section of the tail, which contributes to the majority of the tail mass. In *Siphoviridae* the cylinder of the tail is composed of multiple copies of the tail tube [154,155], whereas in *Myoviridae* phages the tail tube is covered by an outer contractile sheath [152,156,157]. *Podoviridae* phage N4 also has a two-layered tail, albeit short with a non-contractile “sheath” [158]. Initiation of the tail tube polymerization in *Sipho*- and *Myoviridae* phages probably occurs via conformational switching. Without the initiator complex the lambda tail tube protein, gpV, cannot form a tubular structure and exists as a monomer. One of the structural proteins of the bacterial complex, the type VI secretion system, is structurally homologous to lambda gpV. In contrast to gpV, the secretion system homolog does not require an initiator to form the tube. Structural comparison of these homologous tube proteins suggested that a loop-to-helix transition is required to initiate polymerization of gpV [154]. Similarly to lambda gpV, the tail tube protein of *Myoviridae* phage T4 does not assemble into tubes without baseplates [159]. Nevertheless, disassembled tubes of T4 can repolymerize without baseplates [160], probably because of an irreversible conformational switching that occurred during the initial binding of the tube protein to the baseplate. In *Myoviridae* phages after the tail tube is assembled, the tail sheath wraps around it. *Siphoviridae* phage SPP1 has two tail proteins in the ratio 3:1, forming a tail tube, with one of them arising from a translational frame shift [161]. The larger tail protein is predicted to have an additional immunoglobulin-like domain. Although some immunoglobulin-like folds, such as BIG2 domain of phi29, have a function, the roles of others remain unknown. Such domains may have been acquired by phages from hosts [162]. The cylinder of the *Sipho* and *Myoviridae* tail is almost exclusively six-fold symmetric. The two exceptions are the three-fold symmetric tail of *Siphoviridae* bacteriophages phiCbK [163] and T5 [55]. The six-fold symmetry of the tail might be functionally advantageous due to the interaction of the phages with the oligosaccharides which form hexagonal arrays on the outer surface of some bacterial strains [40].

After polymerization of the cylindrical part of the tail, binding of terminator proteins completes the tail assembly [164–166]. Terminator proteins, in turn, interact with the neck proteins attached to the head and mediate the association of the tail with the head. The tail-terminator protein of *Siphoviridae* phage lambda is structurally similar to a *Myoviridae* prophage protein, providing further evidence of the evolutionary relationship of these tails [164]. Moreover, structural and functional comparison of neck, tail tube and tail completion proteins suggest that these proteins evolved from a single ancestral gene [136]. The length of *Sipho-* and *Myoviridae* tails is determined by a tape-measure or ruler protein, also found in cellular complexes such as an injectesome (also called a type III secretion system) and the hook of flagellum [26]. The presence of the ruler protein was first identified in bacteriophage lambda and later in bacteriophage T4 and T5 [167–169]. Presumably, the ruler protein is stretched the entire length of the tail and acts as a scaffold for the polymerization of the tail tube [170]. If a ruler protein extends inside the tail cylinder, it would be about 1000 Å long in lambda or T4 and 5000 Å in bacteriophage G. The bacteriophage T4 tape measure protein consists of 590 residues and, thus, cannot have a helical structure in order to be able to stretch the entire length of the tail. The cryo-EM reconstruction of the T4 and SPP1 tails showed density inside the tail tube, which could be attributed to either the ruler protein or DNA [152,155,156]. The copy number of ruler proteins per phage is unknown, but for the type III secretion system it was determined that only one ruler protein is present per complex [171].

The length of the tail tube in *Myoviridae* phages determines the length of the sheath. In contrast to the tail tube proteins, the tail sheath protein can polymerize into polysheaths in the absence of the tail tube, although 50-fold less efficiently [172,173]. Similarly, capsid protein forms polyheads in the absence of scaffolding proteins. In both cases formation of aberrant structures occurs slower than formation of the viral precursors due to a nucleation barrier. The presence of the tail tube-baseplate complex for the tail sheath or the scaffolding protein for the capsid lowers the nucleation barrier, resulting in a faster assembly. A similar requirement is observed in many viruses, with some viral assemblies primed by nucleic acid [174]. Nucleation complexes are ubiquitous among cellular complexes, like microtubules and actin [175,176]. During the formation of an oligomer, reversible interactions between two subunits sharing one interaction site are unstable. However, if a third subunit binds to the two subunits before they dissociate, the probability of dissociation would be reduced [3]. Hence, after the nucleation barrier is overcome, the polymerization occurs very fast. Likewise, during the formation of the tail sheath no intermediates were observed even when the tube-baseplate complexes were in excess, showing that either complete tail sheaths or only naked tubes were present [172,173]. In addition to nucleating the polymerization of the sheath, the tail tube-baseplate complex induces assembly of the sheath subunits into a helical arrangement that is different to the helical symmetry observed in polysheaths [177]. When assembled on the tail tube, the tail sheath is in its extended conformation with the sheath proteins making contacts to the tail tube as well as to each other. In polysheaths the subunits only make contact with each other and have an arrangement similar to the contracted sheath that occurs after infection. In the contracted form the tail sheath subunits are detached from the inner tube, similarly to polysheaths. The binding of the sheath to the tube does not require nucleation in contrast to the assembly of polysheath and therefore acts as a kinetic trap, inducing assembly of the sheath into the extended higher free-energy structure [157,178].

### 2.7. Assembly Completion

In *Sipho*- and *Myoviridae* phage assembly the completed head and tail are joined spontaneously. In the case of phage T4, the head to tail association is followed by binding of gp wac to the neck region, forming whiskers. The whiskers are essential for attachment of the preassembled long tail fibers [141]. This aligns the fibers along the length of the tail, positioning the proximal part at the baseplate attachment site. Additionally, attachment of fibers through whiskers insures that no fibers would bind to the free tails. The fibers are known to stay in such a retracted position if the conditions (pH and ionic strength) are unfavorable for phage growth, preventing infection [179].

**Figure 2.**
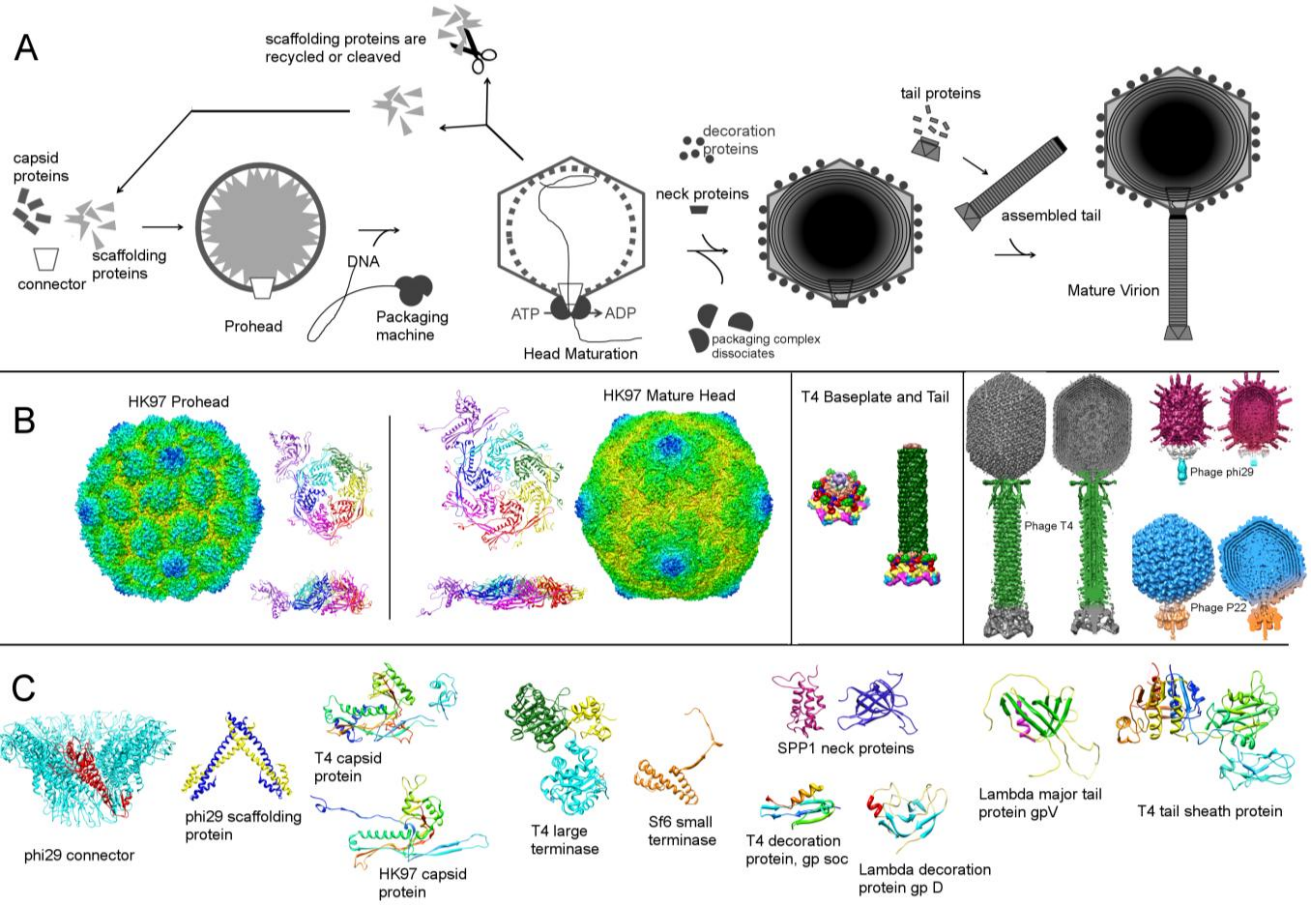
Assembly of dsDNA tailed phages. (**A**) Schematic of the assembly steps. (**B**) Examples of assembly intermediates and mature virions. The left panel shows HK97 prohead and mature head, displaying the whole structure, as well as side and top views of the icosahedral asymmetric units. The middle panel shows the T4 baseplate and tail. The right panel shows T4, phi29 and P22 mature virions, as a surface rendered view and a slab view, displaying the DNA structure. (**C**) Structural proteins involved in the assembly of several phages. The figure was created using the program CHIMERA [180]. The relevant structures were downloaded from the PDB and EMDB databases.

In summary, there are several distinct steps of dsDNA tailed phage assembly: 1. Assembly of a prohead, or a spherical shell of capsid protein filled with scaffolding protein that contains a dodecameric portal. 2. Packaging of DNA using the energy of ATP. 3. Maturation of proheads into angular mature heads. 4. Attachment of the neck and tail proteins or a preassembled tail (Figure 2).

## 3. ssDNA Phages

### 3.1. Microviridae: phiX174

The most studied member of the *Microviridae* family is bacteriophage phiX174. The isometric T = 1 capsid of phiX174 assembles via an empty procapsid intermediate, similarly to dsDNA tailed phages discussed above. In contrast to most dsDNA phages, phiX174 utilizes two scaffolding proteins during assembly: an inner protein B and an outer protein D [53]. The phage assembly is initiated by the association of five capsid proteins (F) with five copies of the internal scaffolding protein (B) into a 9S particle. In turn, the 9S intermediate binds a spike pentamer (G) and a pilot protein (H), forming a 12S particle. Subsequently, binding of twenty external D proteins to a 12S particle results in the formation of an 18S particle, which is a capsomer of a phiX174 procapsid. Association of twelve 18S particles leads to the formation of an icosahedral procapsid [8]. The outer procapsid shell, formed by protein D, is more elaborate than the actual T = 1 capsid underneath. Another bacteriophage that requires an external scaffolding protein during assembly is the satellite phage P4 [181]. In the external scaffolding shell of phiX174, D proteins adopt four different conformations [182], making two types of asymmetric dimers. In a crystal structure D proteins also form asymmetric dimers, which are similar to the two dimers present in the procapsid [183]. The formation of such an asymmetric dimer represents the first step in the assembly of D and is accomplished through conformational switching [183]. Asymmetric dimers are common among cellular enzymes, for example two identical hexokinase molecules form an asymmetric dimer where one of the two subunits binds substrate more tightly than the other [184]. The dimers of phiX174 scaffolding protein D undergo another conformational switch upon tetramerization. The subsequent binding of the D tetramer to the 12S particle induces yet another structural change, allowing twelve 18S subunits to associate. The specific residues involved in the conformational switching of the D protein were identified using structural and mutational investigations [183,185,186].

**Figure 3.**
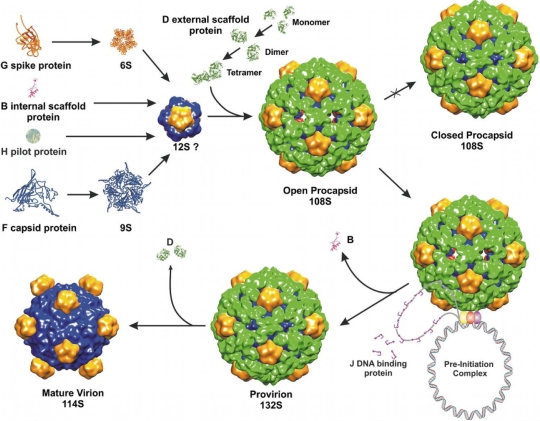
*Microviridae* assembly pathway (reprinted from Bernal *et al.* [189] with permission from Elsevier).

The structure of the phiX174 procapsid was determined by cryo-EM and image reconstruction [187,188]. This structure has 30Å pores at the three-fold axes of the icosahedron and 10Å gaps between the F pentamers, which are bridged together by the external scaffolding D protein shell. This “open” procapsid structure probably represents a true viral precursor. When the same complex was crystallized, it changed into a “closed” procapsid that has a different conformation of F and G proteins, similar to that in a mature virus [182,188]. The “closed” procapsid lacks the three-fold holes and is thought to be an aberrant particle, not a true intermediate state. After assembly, the “open” procapsid is packaged with the ssDNA genome, probably through the three-fold holes, resulting in the exit of the internal scaffolding protein B. Similarly, the internal scaffolding proteins of some dsDNA tailed phages exit from the procapsid though holes in skewed hexameric capsomers, as discussed above. The intermediate assembly state of phiX174, that does not contain B but still contains the external scaffolding protein D, is called “provirion”, which subsequently loses D proteins and matures into a virion [189]. During maturation, instead of the expansion observed in dsDNA tailed phages, the head collapses, as the holes between the F pentamers and the holes at the three-fold axes close and the scaffolding protein dissociates (Figure 3).

### 3.2. Filamentous Phages

Filamentous phages have been widely used as a molecular biology tool since the development of the phage display method [190], which proved indispensable to study protein-protein interactions, modify proteins and select antibodies [191]. The assembly and structure of filamentous phages have been extensively studied [192–196]. The filamentous virion has a 9000 Å-long rod-like structure with a ∼65 Å diameter that contains ssDNA genome. In contrast to DNA bacteriophages discussed above, filamentous phages assemble on the internal membrane of the host cell. The head of the tailed bacteriophage T4 also assembles on the internal membrane of *E. coli*, anchored by a connector chaperon protein, as discussed above. In filamentous bacteriophages, all structural proteins are anchored on the inner membrane of *E. coli*. Moreover, the major capsid protein, pVIII, of filamentous phages, forms a transmembrane helix, but the final virion does not contain any lipids [194]. In contrast to the majority of phages that lyse the infected cell, filamentous phages form adhesion zones and are extruded from the host without killing it.

**Figure 4.**
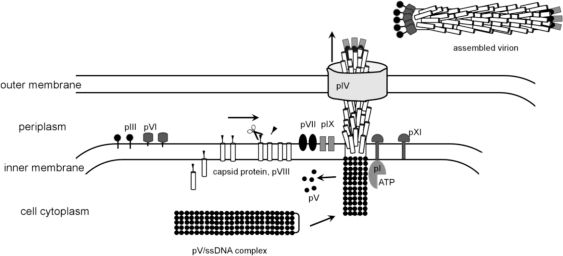
Model for the assembly and extrusion of filamentous phages. The figure was created based on a figure by Russel and Model [196].

Filamentous phages belong to *Inoviridae* family, represented by M13, fd and f1 as most studied members that infect *E. coli*. Infectious virions are assembled from five different structural proteins. There are about 2,700 copies of the major capsid protein that form a five-start helical rod [197–200], capped by pentamers of pIII and pVI on one side and pVII and pIX on the other [201,202]. In some filamentous phages, e.g., f1, the capsid protein has a leader sequence that is cleaved by a cellular peptidase after membrane insertion [203]. Three non-structural proteins, pI, pIV and pXI, mediate assembly and release of the phage from the host cell. The assembly is initiated when the ssDNA binding protein pV displaces the DNA replication machinery. Next, the pI protein interacts with the 32 bp DNA leader sequence and mediates the DNA interaction with capsid protein, removing pV. It has been suggested that this process requires ATP hydrolyses. Because pI has a nucleotide-binding motif, it was proposed to be a motor [193]. If this is indeed the case this would be the second use of an ATP-driven motor employed in bacteriophage assembly, with the first motor being the phage packaging machine. Prior to DNA binding, the capsid protein is probably associated with minor proteins pVII and pIX, which are required for assembly initiation [202]. It is possible that they trigger a conformational switch in the capsid protein, inducing it to polymerize around DNA. How the capsid protein transitions from the lipid membrane into the rod that does not contain any lipids is unknown. The length of the rod is determined by the length of DNA. In contrast to TMV, where three bases fit into a defined groove of the capsid protein, there is no specific protein/DNA association in filamentous phages. The length of packaged DNA can be altered by the number of charged residues of the capsid protein [204,205]. The terminal cap proteins pIII and pVI bind last and terminate the assembly. Similar to the tail assembly termination proteins of dsDNA phages, the absence of pIII and pVI results in formation of polyphages. The virions exit through the adhesion zones, formed by two non-structural proteins pI and pXI (Figure 4). Another viral protein, pIV, was shown to assemble into 10–12 subunit oligomers that probably form pores in the outer membrane of the cell [206,207]. Homologs of pIV were found in both type II and type III bacterial secretion systems. The location of gene IV in the genomes of several different filamentous phages is not conserved, in comparison with other phage proteins, suggesting that filamentous phages could have acquired the pIV gene from a host [193].

## 4. Lipid Containing Phages

The two groups, *Tectiviridae* and *Cystoviridae*, described below assemble via an empty capsid shell, similar to dsDNA tailed phages and ssDNA isometric phages.

**Figure 5.**
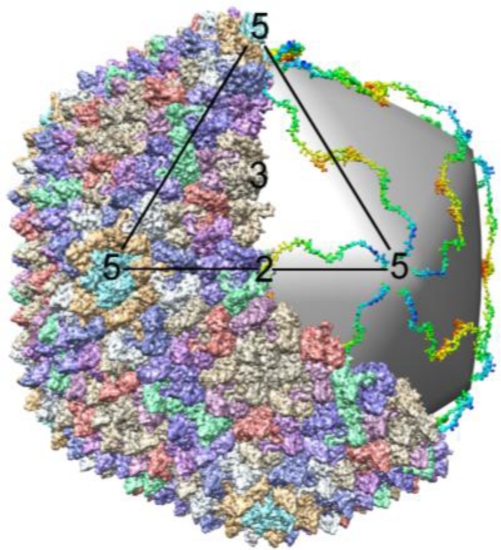
Structure of PRD1 virion. A portion of the capsid protein was removed to show the mesh underneath made by 60 copies of tape measure protein p30. The figure was created using the program CHIMERA [180]. The relevant PDB entry 1W8X was downloaded from the PDB databank.

The most studied member of the *Tectiviridae* family is bacteriophage PRD1. The crystal structure of PRD1 was determined by X-ray crystallography, showing the arrangement of capsid proteins that surround the membrane sack and dsDNA genome [208,209]. Four of eighteen viral structural proteins are icosahedrally arranged and, therefore, visible in the X-ray crystal structure. Although phages from the *Tectiviridae* group do not have a permanent or stable tail structure, a non-permanent tail is produced upon infection [210,211]. Furthermore, PRD1 has a special five-fold vertex with a different protein composition to the proteins at other five-fold axes [212] and carries the packaging ATPase [213]. However, this unique vertex has not been shown to be the same as the vertex used for infection, but is utilized during packaging [214]. Therefore, in contrast to the tailed dsDNA phages, the PRD1 packaging enzyme is a structural protein and does not dissociate after the assembly is complete [17,212]. The rate of PRD1 packaging *in vitro* was calculated to be more than 340 bp/s [215], which is comparable with that of some dsDNA tailed phages (e.g., 350 bp/s for SPP1 [216]). Bacteriophage T4 can package with a rate of up to 2000 bp/s [217]), whereas dsRNA phages translocate RNA at a much slower rate of about 30 bp/s [218]. Although PRD1 has no temporary scaffolding, its membrane is coated by the structural protein p30. This glue protein mediates the assembly of the coat protein p3 into correct pseudo T = 25 quasi-symmetry and cements capsomers together (Figure 5) [208]. A similar, but external, network of scaffolding proteins is formed by protein Sid of bacteriophage P4, a satellite phage of P2 [181,219]. No expansion of the PRD1 capsid was detected after DNA packaging, although expansion of the viral membrane was observed [220]. There are many similarities between phage PRD1 and adenovirus, including the topology of the capsid protein jelly-roll fold, capsomer arrangement and replication priming by a protein. These similarities suggest that although these viruses infect two different domains of life they might be evolutionary related [221,222]. Additionally, recent structures of adenovirus by X-ray crystallography [223] and atomic resolution cryo-EM [224] showed that one of the adenovirus cement proteins, although structurally different from PRD1 p30, also forms a net underneath the capsid protein [224]. This adenoviral cement protein affects the amount of DNA packaged by the virus [225,226] and might be a functional equivalent of the PRD1 tape measure protein p30.

**Figure 6.**
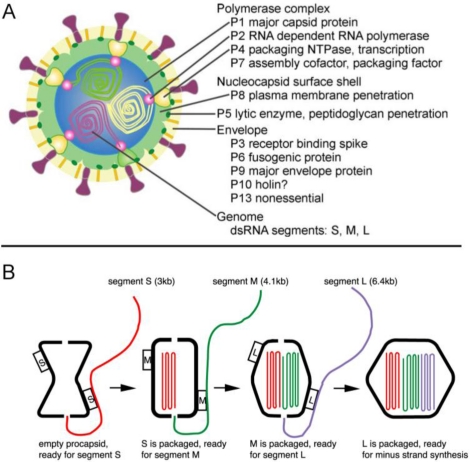
(**A**) A diagram showing the phi6 virion architecture (reprinted from [228] with permission from Elsevier). (**B**) Genome packaging in dsRNA phages (reprinted from Figure 6 in Mindich *et al.* [16] with permission from Elsevier).

Another group of lipid-containing viruses that package their nucleic acid into the preformed capsid shell belong to the *Cystoviridae* family of phages and have a segmented dsRNA genome. The most studied members of this group are bacteriophages phi6 and phi12. The procapsid of *Cystoviridae* consists of 120 copies of the capsid protein (p1), about 14 copies of RNA polymerase (p2), located at the five-fold vertices [227], a packaging enzyme p4 and its cofactor p7 (Figure 6A). The procapsid is not spherical and has deeply recessed vertexes [220]. The genome is packaged into the procapsid, somewhat similarly to the DNA phages discussed above. Because the genome is segmented, packaging is sequential. The (+) strands of the S, M and L segments are packaged in strict order, probably determined by the binding site for the RNA strand on the capsid surface. Once S, the first segment, is packaged another binding site appears for the next strand to be packaged, which continues until the third segment is internalized. After all (+) stands are packaged the (−) strands are synthesized inside the capsid by the polymerase (Figure 6B) [16,228]. The packaging is accompanied by capsid expansion resulting in elimination of surface depressions. Both RNA polymerase and the packaging enzyme are structural proteins [229]. The phi6 packaging enzyme can use any nucleotide for packaging in contrast to dsDNA tailed phages, which have specificity for ATP. A mechanism of the RNA translocation was proposed based on the X-ray structure of the NTPase [101]. In contrast to PRD1, where ATPase is located only on one special vertex, the phi6 ATPase (p4) is present on more than one vertex [229]. Upon completion of assembly and packaging of phi6, the procapsid is coated with another protein, p8, that forms a T = 13 icosahedral shell. This outer shell has a different arrangement in related phages, such as phi12 and phi8 [230,231]. The assembly is completed when the virus has been surrounded by a lipid membrane containing four integral membrane proteins [228]. The formation of the lipid envelope does not involve budding and is not understood.

## 5. ssRNA Icosahedral Phages

The single-stranded RNA phages such as MS2, Qβ, fr, GA, R17, f2 and phiCb5 form the *Leviviridae* family. The capsid protein of ssRNA phages has a unique fold, specific only to this phage group. Ninety dimers of the capsid protein are arranged into a T = 3 icosahedral lattice [232–236]. Additionally, the virion contains one copy of a maturation protein, or A protein and viral ssRNA genome. Similar to some ssRNA plant viruses, a large proportion of the ssRNA is icosahedrally ordered [174]. The A protein mediates phage attachment to pili and was suggested to initiate assembly of the coat protein around the folded RNA [237]. Even without the genome, the coat protein assembles into virus-like particles, although this requires higher coat protein concentration. In the T = 3 capsid there are two quasi-equivalent coat protein dimers, both of which are required for efficient assembly. The ssRNA induces the conformational switching of the capsid protein dimer during assembly [238,239]. In the assembly of *Leviviridae* phiCb5 phage, three RNA bases interact with the two capsid subunits from one dimer as well as one subunit from a neighboring dimer, facilitating capsid formation and stabilizing the final structure [240]. Co-interaction of capsid protein with the RNA is critical during assembly, similar to the interactions between scaffold and capsid proteins in dsDNA phages.

## 6. Concluding Remarks

The majority of structural information about phage assembly has been accumulated by studying individual viral components. Although the dynamics of the viral assembly processes has been investigated with biophysical methods [241–244], most of current models of dynamic processes are based on investigations of stable intermediates, captured by slowing the assembly reactions *in vitro*. Analysis of transient dynamic intermediates will provide new insights into molecular mechanisms of phage assembly. Visualization of the conformational changes, like capturing the rearrangement of the capsid proteins during the capsid maturation, might soon be possible with the development of live high-speed imaging techniques [245].

Despite great morphological and genetic diversity of phages, there are extensive structural similarities in the component proteins from which the whole virus is assembled and that govern the viral life cycle. As has frequently been observed, the three-dimensional structure of proteins is perhaps the most conserved aspect of evolution [246]. The most highly conserved structures relate to the most basic biological functions, such as ATP driven molecular motors and the capsid structures of many phage families. The implication of the conserved capsomer structure with either a HK97 or a jelly-roll fold is that phages and, hence, bacteria have co-evolved and that this coexistence has provided the means for gene transfer and the subsequent evolutionary modifications in all kingdoms of life.

## Figures and Tables

**Table 1.** Phages discussed in the current review.

DNA genome						RNA genome	
dsDNA				ssDNA		dsRNA lipid containing *Cystoviridae*	ssRNA *Leviviridae*
*Caudovirales* (dsDNA tailed phages)			dsDNA lipid containing *Tectiviridae*	Filamentous *Inoviridae*	Icosahedral *Microviridae*		
*Podoviridae*	*Myoviridae*	*Siphoviridae*	**PRD1**	**M13, fd, f1**	**phiX174**	**phi6, phi8, phi12**	**MS2, Qβ, fr, GA, R17, f2, phiCb5**
**P22, T7, phi29, N4**	**T4, Mu, G, P2/P4**	**Lambda, SPP1, HK97, T5, p2**					
